# The Hygiene of Old Age, with Special References to Prostatic Hypertrophy and Secondary Disease of the Bladder

**Published:** 1881-05

**Authors:** Reuben A. Vance

**Affiliations:** Cincinnati, Ohio


### THE HYGIENE OF OLD AGE, WITH SPECIAL REFERENCES TO PROSTATIC HYPERTROPHY AND SECONDARY DISEASE OF THE BLADDER.

By REUBEN A. VANCE, M D., of Cincinnati, Ohio.

Read before the Cincinnati Academy of Medicine.

The subject chosen as a text for my remarks this evening is one that cannot be treated in part only on such an occasion as this. I have, therefore, determined to limit my remarks to the indications and effects of prostatic hypertrophy, and the steps to be taken for its relief. Curtailed as is the subject by these restrictions, it is, nevertheless, too voluminous for thorough treatment in the time at my disposal. Therefore, if I but outline to you a few of the more important facts bearing upon senile enlargement of the prostate body, and review such of the morbid anatomical conditions of the urinary apparatus as I deem of interest in this connection, and then allude to the method of relief I consider of vital importance, I shall accomplish all I think it advisable to attempt.

The normal prostate, let me presume, is shaped like a chestnut, and, longitudinally, is an inch and a half from base to apex, and one and three-quarters transversely near the base, and seven eights of an inch in thickness at the apex, while in weight it is from four and a half to five drahms. Now, while marked excess in either measurement or weight denotes hypertrophy, alteration in form is a far more certain criterion by which to judge. Nevertheless, there are many cases in which bladder-disease developes as a consequence of senile enlargement of the prostate, in which physical exploration fails to yield any evidence of increased bulk or modified form on the part of the prostate body.

Sir Henry Thompson, who, conjointly with Dr. Messer, dissected the prostate in about 200 subjects over 55, found that about one in three exhibited some enlargement of that organ, but only one of these out of seven had had symptoms of the complaint. According to Thompson, the period of life between 55 and 65 is that during which the affection most commonly begins to be developed. He has never met with an instance of hypertrophy of the prostate at so early a period as 50 years of age, while on the other hand he says it appears rarely to commence after 80 or 85. He thinks that the man who has escaped enlargement of the prostate at 65 will never be troubled with it In senile hypertrophy there is an augmentation of the muscular and connective tissues of the prostate, with little, if any, increase of the glandular element. There is increase of volume, weight, and density, with modifications of form. The enlargement may be general or partial.

Common as is prostatic hypertrophy, and well defined as are the symptoms accompanying its advanced stages, there are few affections more obscure in their early manifestations. A few months ago a well-known gentleman of this city stopped me on the street with the remark that he had been intending to call and see me for some time; that his kidneys were troubling him, and he wanted something that would relieve the distress from which he was suffering. A question or two speedily revealed the nature of the case. For months he had been compelled to get up two or three times of a night to relieve his bladder, and he was now conscious that each act of micturition, although prolonged and difficult, was of little avail so far as a free evacuation of the urine was concerned. The urine was clear; it never had contained blood; but there was, at the time of my first interview with the patient, an almost constant call to urinate, and each act freed but a small quantity of urine. No amount of explantion was sufficient to make him appreciate the true nature of the case. He had an all-sufficient explanation for the phenomena troubling him so greatly; this was, "irritability of the bladder," alternating with "congestion of the kidneys." If the nature of senile enlargement of the prostate was spoken of, and the fact that his age—he was in his 49th year—predisposed him to it, and, furthermore, if his attention was called to the fact that the frequency of micturition from which he suffered was due to retention and repletion—a morbid degree of retention of urine, owing to his inability to evacuate more than a small quantity, thus leaving a certain amount of residual urine in the bladder, which increased week by week—he was sure that the contrary was the case, for he knew that he was passing more water than usual. According to his views of the matter—one supported by the authority of his family physician—his kidneys were congested enough to cause hyper secretion of the urine, and his bladder was so irritated that it would retain but a few tablespoonsful at a time. To try to make this patient believe that his bladder was water logged, so to speak, that he was carrying about with him from a pint to two quarts of water, each act of micturition but dicharging a gill or so of urine, the organ never emptying itself completely, but on the contrary gradually falling into a diseased state from the continuance of the unnatural conditions under which it was placed, was mere waste of time. The last act in the preliminary stage of this case occurred within ten days of the interview referred to. He called and announced that something must be done for his relief, for he was in a deplorable plight; that his bladder, "which had been so irritable as to retain but a small amount of urine at a time, for months," had now utterly failed him; that for a week water had been dribbling from him at night, while sleeping, while for the last forty-eight hours it ran away from him, drop by drop, sleeping or waking. Upon passing a catheter, twenty-seven ounces of urine were withdrawn; finding the bladder distended still, the catheter was removed, and the patient informed of the necessity for prolonged treatment.

This patient's experience is typical of that of many who suffer from this complaint. In the very beginning the individual may be conscious of nothing but an unusual degree of reluctance—if I may so express it—on the part of the bladder to part with its contents. The patient experiences a call to urinate; he places himself in position to respond to nature's demand, but so soon as he does, the power of the bladder to empty itself seems temporarily impaired. The experienced surgeon sees in this condition the influence of an abnormal prostate; far different is it with the patient. Without he, by chance, secures competent professional advice, the patient never knows the true nature of his disease, or even that it is a disease, until the condition of his bladder forces him to seek surgical aid. A want of power to promptly empty the bladder in a man between fifty and sixty years of age, almost invariably indicates prostatic enlargement. If, in addition to this delay in emptying the bladder, there is either an increase in the frequency of the call to urinate, or a diminution in the quantity of urine passed, this condition is denoted. Frequency of micturition, a diminution in the quantity of urine voided, a failure or marked impairment of the individual's ability to empty the bladder promptly and satisfactorily, indicate commencing obstruction from senile enlargement of the prostate. In a word, when a man beyond forty-five years of age experiences an increase in the frequency of his calls to empty the bladder, or has his attention drawn to that organ by a delay in emptying it, or an inability to accelerate the flow of urine after it starts, he should suspect prostatic hypertrophy.

The anatomical condition accompanying prostatic hypertrophy, preventing complete evacuation of the bladder, is very simple and quite devoid of all danger, provided proper hygienic measures are adopted from the first. In the beginning but a minute amount of urine may remain in the bladder. It is not improbable that, in many cases, accident initiates this condition. In order to empty the bladder, time is necessary. Circumstances which prevent the patient giving the requisite attention to this function may be the means of developing secondary disease of the bladder. A patient with commencing hypertrophy of the prostate—a lawyer—was engaged in an exciting trial, and the day he addressed the jury he neglected the proper evacuation of his bladder; that at night the urine dribbled away while he was asleep; the next morning, after passing a catheter, I discharged a quart and a half of urine, and found the bladder completely atonied. If individuals suffering from senile enlargement of the prostate could be made to appreciate the fact that the mere hypertrophy of that organ is a a matter of secondary consideration, while the condition of the bladder is of vital moment, fewer sad cases of secondary vesical disease would be encountered.

I have said little, as yet, of that condition in which these cases all result: distension of the bladder with overflow of urine. Every surgeon knows too well how commonly these cases are mistaken for the very opposite state; how the sufferer will argue that he cannot have anything but incontinence, for the self-evident reason that he is constantly passing water. Yet, in truth, his condition is but the final stage of the cases to which I have been alluding. Day by day, and little by little, there is a slow accumulation of urine in the gradually distending bladder. At first the patient, finding it tedious to stand in the water-closet and completely empty his bladder, discharges only enough to relieve himself for the time. The ounce or two of urine thus left by negligence may not be voluntarily voided at the next act; in this way the seeds of urinary decomposition and bladder diseases are planted. In one case the quantity of urine increases from day to day, until finally the bladder becomes full and its walls atonied; in another case the retained urine undergoes decomposition, and inflammation of the lining membrane of the bladder is induced; in both, the resulting disease can be traced to the neglect of complete evacuation of the bladder. Strange to say, if the practitioner is careful to save and measure the urine daily passed by such a patient, the amount voided in the twenty-four hours will be found fully equal to the ordinary discharge of a healthy individual in the same interval of time. It does seem as if the local irritation had some effect in increasing the quantity of urine secreted; this is especially true as regards the night as distinguished from the day time. Such a patient will be compelled to get out of bed and use the night-vessel from five to thirty times between bedtime and morning. While the amount voided on each occasion may not be large, yet upon taking the whole quantity discharged in the twenty-four hours and comparing it with that usually passed daily, in health, there will be no material difference. This partially explains the incredulity of certain patients when informed of the nature of their disease. Any intelligent man can understand his surgeon when the latter tells him that his bladder is irritable and impatient when but a minute amount of urine is present ; that his frequent calls to micturate and trouble in emptying the bladder arise from vesical irritability and urinary incontinence. Quite different is it when an endeavor is made to explain the complex conditions arising from prostatic obstruction, muscular atony and vesical catarrh. The patient shrinks from an instrumental examination, and is loth to have a catheter introduced, even for diagnostic purposes.

Yet, upon the proper employment of this last instrument depends the prevention of secondary disease of the vesical cavity in individuals suffering from prostatic enlargement, and the relief of those patients who have been so unfortunate as to have atony of the muscular walls, or catarrh of the lining membrane of the bladder ensue as a consequence of prior diseases of the prostate body. Just so soon as an individual of the male sex, who has passed his forty-fifth year, finds himself compelled to micturate more frequently than usual—especially at night, and under circumstances excluding obstructive disease of the urethra —surgical advice should be taken, and a catheter employed. If a catheter is then passed—and it should be used immediately after the patient has voided all the urine he can voluntarily discharge—and a quantity of residual urine withdrawn, the surgeon has an unmistakable guide bo h as to diagnosis and treatment. That process of urinary retention has been inaugurated, which, if not relieved, will certainly result in either atony of the vesical walls and indefinite distensibility, or in contraction of the bladder and partial hypertrophy of the muscular layers. The details of the measures which should be adopted in connection with the use of the catheter in these cases I need not dwell upon ; suffice it to say, that a few weeks' judicious employment of that instrument will relieve even the most unpromising cases, and permanently cure the majority of patients with commencing bladder disease. I shall also dismiss, with a word, the danger attendant upon the injudicious employment o the catheter. Sir Benjamin C Brodie long since pointed out a peculiar class of patients in whom the complete evacuation of the urine by the catheter, at one time, was apt to be followed by serious results. This danger can always be obviated by removing but a part of the residual urine at one time: in order to do this, the catheter should be introduced and the bladder emptied but twice a day, for three days. The point upon which I desire to lay special stress, is the necessity for immediate resort to the catheter so soon as the early symptoms of prostatic obstruction declare themselves, and its use sufficiently often thereafter to empty the bladder at least once daily, and thus prevent urinary accumulation and vesical disease. By adopting this course the great number of cases of bladder disease in old men may be reduced to the minimum, and the profession afford most efficient service in advancing the hygiene of old age, with special reference to those secondary affections of the vesical cavity which have their origin in prostatic hypertrophy.

